# Feeling the future: A meta-analysis of 90 experiments on the anomalous anticipation of random future events

**DOI:** 10.12688/f1000research.7177.2

**Published:** 2016-01-29

**Authors:** Daryl Bem, Patrizio Tressoldi, Thomas Rabeyron, Michael Duggan

**Affiliations:** 1 Cornell University, New York, NY, 10011, USA; 2 Università di Padova, Padova, 35122, Italy; 3 Université de Nantes, Nantes, 44300, France; 4 University of Edinburgh, Edinburgh, Scotland, EH8 9YL, UK; 5 Nottingham Trent University, Nottingham, England, NG1 4BU, UK

**Keywords:** precognition, psi, ESP, retrocausation, retro-priming, parapsychology

## Abstract

In 2011, one of the authors (DJB) published a report of nine experiments in the *Journal of Personality and Social Psychology* purporting to demonstrate that an individual’s cognitive and affective responses can be influenced by randomly selected stimulus events that do not occur until after his or her responses have already been made and recorded, a generalized variant of the phenomenon traditionally denoted by the term *precognition*. To encourage replications, all materials needed to conduct them were made available on request. We here report a meta-analysis of 90 experiments from 33 laboratories in 14 countries which yielded an overall effect greater than 6 sigma, *z* = 6.40, *p *= 1.2 × 10 ^-10  ^with an effect size (Hedges’ *g*) of 0.09. A Bayesian analysis yielded a Bayes Factor of 5.1 × 10 ^9^, greatly exceeding the criterion value of 100 for “decisive evidence” in support of the experimental hypothesis. When DJB’s original experiments are excluded, the combined effect size for replications by independent investigators is 0.06, *z* = 4.16, *p* = 1.1 × 10 ^-5^, and the BF value is 3,853, again exceeding the criterion for “decisive evidence.” The number of potentially unretrieved experiments required to reduce the overall effect size of the complete database to a trivial value of 0.01 is 544, and seven of eight additional statistical tests support the conclusion that the database is not significantly compromised by either selection bias or by intense “ *p*-hacking”—the selective suppression of findings or analyses that failed to yield statistical significance. *P*-curve analysis, a recently introduced statistical technique, estimates the true effect size of the experiments to be 0.20 for the complete database and 0.24 for the independent replications, virtually identical to the effect size of DJB’s original experiments (0.22) and the closely related “presentiment” experiments (0.21). We discuss the controversial status of precognition and other anomalous effects collectively known as *psi*.

In 2011, the *Journal of Personality and Social Psychology* published an article by one of us (DJB) entitled “Feeling the Future: Experimental Evidence for Anomalous Retroactive Influences on Cognition and Affect” ( Bem, 2011). The article reported nine experiments that purported to demonstrate that an individual’s cognitive and affective responses can be influenced by randomly selected stimulus events that do not occur until after his or her responses have already been made and recorded, a generalized variant of the phenomenon traditionally denoted by the term *precognition*. The controversial nature of these findings prompted the journal’s editors to publish an accompanying editorial justifying their decision to publish the report and expressing their hope and expectation that attempts at replication by other investigators would follow ( Judd & Gawronski, 2011).

To encourage replications from the beginning of his research program in 2000, Bem offered free, comprehensive packages that included detailed instruction manuals for conducting the experiments, computer software for running the experimental sessions, and database programs for collecting and analyzing the data. As of September 2013, two years after the publication of his article, we were able to retrieve 69 attempted replications of his experiments and 11 other experiments that tested for the anomalous anticipation of future events in alternative ways. When Bem’s experiments are included, the complete database comprises 90 experiments from 33 different laboratories located in 14 different countries.

Precognition is one of several phenomena in which individuals appear to have access to “nonlocal” information, that is, to information that would not normally be available to them through any currently known physical or biological process. These phenomena, collectively referred to as *psi*, include *telepathy,* access to another person’s thoughts without the mediation of any known channel of sensory communication; *clairvoyance* (including a variant called *remote viewing*), the apparent perception of objects or events that do not provide a stimulus to the known senses; and *precognition*, the anticipation of future events that could not otherwise be anticipated through any known inferential process.

Laboratory-based tests of precognition have been published for nearly a century. Most of the earlier experiments used forced-choice designs in which participants were explicitly challenged to guess on each trial which one of several potential targets would be randomly selected and displayed in the near future. Typical targets included ESP card symbols, an array of colored light bulbs, the faces of a die, or visual elements in a computer display. When a participant correctly predicted the actual target-to-be, the trial was scored as a hit, and performance was typically expressed as the percentage of hits over a given number of trials.

A meta-analysis of all forced-choice precognition experiments appearing in English language journals between 1935 and 1977 was published by Honorton & Ferrari (1989). Their analysis included 309 experiments conducted by 62 different investigators involving more than 50,000 participants. Honorton and Ferrari reported a small but significant hit rate, Rosenthal effect size z/√n = .02, Stouffer *Z* = 6.02, *p* = 1.1 × 10 ^-9^. They concluded that this overall result was unlikely to be artifactually inflated by the selective reporting of positive results (the so-called file-drawer effect), calculating that there would have to be 46 unreported studies averaging null results for every reported study in the meta-analysis to reduce the overall significance of the database to chance.

Just as research in cognitive and social psychology has increasingly pursued the study of affective and cognitive processes that are not accessible to conscious awareness or control (e.g., Ferguson & Zayas, 2009), research in psi has followed the same path, moving from explicit forced-choice guessing tasks to experiments using subliminal stimuli and implicit or physiological responses. This trend is exemplified by several “presentiment” experiments, pioneered by Radin (1997) and Bierman ( Bierman & Radin, 1997) in which physiological indices of participants’ emotional arousal are continuously monitored as they view a series of pictures on a computer screen. Most of the pictures are emotionally neutral, but on randomly selected trials, a highly arousing erotic or negative image is displayed. As expected, participants show strong physiological arousal when these images appear, but the important “presentiment” finding is that the arousal is observed to occur a few seconds before the picture actually appears on the screen—even before the computer has randomly selected the picture to be displayed.

The presentiment effect has now been demonstrated using a variety of physiological indices, including electrodermal activity, heart rate, blood volume, pupil dilation, electroencephalographic activity, and fMRI measures of brain activity. A meta-analysis of 26 reports of presentiment experiments published between 1978 and 2010 yielded an average effect size of 0.21, 95% CI = [0.13, 0.29], combined *z* = 5.30, *p* = 5.7 × 10 ^-8^. The number of unretrieved experiments averaging a null effect that would be required to reduce the effect size to a trivial level was conservatively calculated to be 87 ( Mossbridge *et al.*, 2012; see also, Mossbridge *et al.*, 2014). A critique of this meta-analysis has been published by Schwarzkopf (2014) and the authors have responded to that critique ( Mossbridge *et al.*, 2015).

 Bem’s experiments can be viewed as direct descendants of the presentiment experiments. Like them, each of his experiments modified a well-established psychological effect by reversing the usual time-sequence of events so that the participant’s responses were obtained before the putatively causal stimulus events occurred. The hypothesis in each case was that the time-reversed version of the experiment would produce the same result as the standard non-time-reversed experiment. Four well-established psychological effects were modified in this way. (See Bem (2011) for more complete descriptions of the experimental protocols.)

## Precognitive approach and avoidance

Two experiments tested time-reversed versions of one of psychology’s oldest and best known phenomena, the Law of Effect ( Thorndike, 1898): An organism is more likely to repeat responses that have been positively reinforced in the past than responses that have not been reinforced. Bem’s time-reversed version of this effect tested whether participants were more likely to make responses that would be reinforced in the near future. On each trial of the first experiment (“Precognitive Detection of Erotic Stimuli”), the participant selected one of two curtains displayed side-by-side on a computer screen. After the participant had made a choice, the computer randomly designated one of the curtains to be the reinforced alternative. If the participant had selected that curtain, it opened to reveal an erotic photograph and the trial was scored as a hit; if the participant had selected the other curtain, a blank gray wall appeared and the trial was scored as a miss. In a second experiment (“Precognitive Avoidance of Negative Stimuli”) a trial was scored as a hit if the participant selected the alternative that avoided the display of a gruesome or unpleasant photograph.

## Retroactive priming

In recent years, priming experiments have become a staple of cognitive social psychology ( Klauer & Musch, 2003). In a typical affective priming experiment, participants are asked to judge as quickly as they can whether a photograph is pleasant or unpleasant and their response time is measured. Just before the picture appears, a positive or negative word (e.g., *beautiful*, *ugly*) is flashed briefly on the screen; this word is called the prime. Individuals typically respond more quickly when the valences of the prime and the photograph are congruent (both are positive or both are negative) than when they are incongruent. In the time-reversed version of the procedure, the randomly-selected prime appeared after rather than before participants judge the affective valence of the photograph.

## Retroactive habituation

When individuals are initially exposed to an emotionally arousing stimulus, they typically have a strong physiological response to it. Upon repeated exposures the arousal diminishes. This *habituation* process is one possible mechanism behind the so-called “mere exposure” effect in which repeated exposures to a stimulus produce increased liking for it ( Bornstein, 1989; Zajonc, 1968). It has been suggested that if a stimulus is initially frightening or unpleasant, repeated exposures will render it less negatively arousing and, hence, it will be better liked after the exposures—the usual mere exposure result—but if the stimulus is initially very positive, the repeated exposures will render it boring or less positively arousing and, hence, it will be *less* well liked after the exposures ( Dijksterhuis & Smith, 2002).

In two time-reversed habituation experiments, pairs of negative photographs matched for equal likeability or pairs of erotic photographs similarly matched were displayed side by side on the screen and the participant was instructed on each trial to indicate which one he or she liked better. After the preference was recorded, the computer randomly selected one of the two photographs to be the habituation target and flashed it subliminally on the screen several times. The hypothesis was that participants would prefer the habituation target on trials with negative photographs but would prefer the nontarget on trials with erotic photographs.

The three time-reversed effects described above can be viewed as conceptual replications of the presentiment experiments in that all these experiments assessed affective responses to emotionally arousing stimuli before those stimuli were randomly selected and displayed. Whereas presentiment experiments assess physiological responses, Bem’s experiments assessed behavioral responses. Even the photographs used in the two kinds of experiments were drawn primarily from the same source, the International Affective Picture System (IAPS; Lang & Greenwald, 1993), a set of more than 800 digitized photographs that have been rated for valence and arousal.

## Retroactive facilitation of recall

A commonplace phenomenon of memory is that practicing or rehearsing a set of verbal items facilitates their subsequent recall. Two of Bem’s time-reversed experiments tested whether rehearsing a set of words makes them easier to recall even if the rehearsal takes place after the recall test is administered. Participants were shown 48 common nouns one at a time on the computer screen. They were then given a (surprise) recall test in which they were asked to type out all the words they could recall, in any order. After the participant completed the recall test, the computer randomly selected half the words to serve as practice words and had participants rehearse them in a series of practice exercises. The hypothesis was that this practice would “reach back in time” to facilitate the recall of these words and, thus, participants would recall more of the to-be-practiced words than the control non-practiced words.

This protocol is methodologically and conceptually quite different from the three time-reversed protocols described above. In those, participants were required to make quick judgments on each trial with no time to reflect on their decisions. The sequence of events within each trial occurred on a time scale of milliseconds and the putatively causal stimulus appeared immediately after each of the participant’s responses. In terms of Kahneman’s (2011) dual-mode theory of cognition—as described in his book, *Thinking, Fast and Slow—*these experiments required cognitive processing characteristic of System 1, “Fast Thinking” (also see Evans, 2008, and Evans & Stanovich, 2013).

In contrast, the retroactive facilitation-of-recall protocol confronted participants with a single extended cognitive task that occurred on a time scale of minutes: Presenting the initial list of words took 2-1/2 minutes; the recall test took up to 5 minutes; and the post-test practice exercises took approximately 7 minutes. This allowed participants time to implement deliberate conscious strategies involving working memory, active rehearsal, and verbal categorization, all cognitive processes characteristic of System 2, “Slow Thinking.”

Across all his experiments, Bem reported a mean effect size ( *d*) of 0.22, with a Stouffer *Z* of 6.66, *p* = 2.68 × 10 ^-11^ ( Bem *et al.*, 2011).

Bem’s experiments have been extensively debated and critiqued. The first published critique appeared in the same issue of the journal as Bem’s original article ( Wagenmakers *et al.*, 2011). These authors argued that a Bayesian analysis of Bem’s results did not support his psi-positive conclusions and recommended that all research psychologists abandon frequentist analyses in favor of Bayesian ones. Bem *et al.* (2011) replied to Wagenmakers *et al.*, criticizing the particular Bayesian analysis they had used and demonstrating that a more reasonable Bayesian analysis yields the same conclusions as Bem’s original frequentist analysis. In a similar critique, Rouder & Morey (2011) also advocated a Bayesian approach, criticizing the analyses of both Bem and Wagenmakers *et al.* Rather than continuing to debate this issue in the context of Bem’s original experiments, we here analyze the current database with both a frequentist analysis and the specific Bayesian analysis recommended by Rouder and Morey for meta-analyses.

Recently, Judd *et al.* (2012) have argued that psychologists should start treating stimuli statistically as a random factor the same way we currently treat participants. As they acknowledge, this would constitute a major change in practice for psychologists. To illustrate, they re-analyzed several published datasets from psychological journals, including one of Bem’s retroactive priming results, showing that when stimuli are treated as a random factor the results are statistically weaker than reported in the original articles. They conclude that “As our simulations make clear, in many commonly used designs in social cognitive research, a likely consequence of only treating participants as a random effect is a large inflation of Type I statistical errors, well above the nominal .05 rate (p. 12).”

Francis (2012) and Schimmack (2012) take a different tack. Instead of arguing that Bem’s results are weaker than he reports, they argue that, on the contrary, his results are actually too good to be true. That is, given the statistical power of Bem’s effects, it is unlikely that eight of his nine experiments would have achieved statistical significance, implying that there is a hidden file-drawer of experiments or failed statistical analyses that Bem failed to report.

In his own discussion of potential file-drawer issues, Bem (2011) reported that they arose most acutely in his two earliest experiments (on retroactive habituation) because they required extensive pre-experiment pilot testing to select and match pairs of photographs and to adjust the number and timing of the repeated subliminal stimulus exposures. Once these were determined, however, the protocol was “frozen” and the formal experiments begun. Results from the first experiment were used to rematch several of the photographs used for its subsequent replication. In turn, these two initial experiments provided data relevant for setting the experimental procedures and parameters used in all the subsequent experiments.

As Bem’s explicitly stated in his article, he omitted one exploratory experiment conducted after he had completed the original habituation experiment and its successful replication. It used supraliminal rather than subliminal exposures. He noted that this fundamentally alters the participant’s phenomenology of the experiment, transforming the task into an explicit ESP challenge and thereby undermining the very rationale for using an implicit response measure of psi in the first place. Even that experiment was not left languishing in a file drawer, however, because he had reported and critiqued it at a meeting of the Parapsychological Association ( Bem, 2003).

With regard to unreported data analyses, Bem analyzed and reported each experiment with two to four different analyses, demonstrating in each case that the results and conclusions were robust across different kinds of analyses, different indices of psi performance, and different definitions of outliers. Following standard practice, however, he did not treat stimuli as a random factor in his analyses.

In his own critique, Francis (2012) remarks that “perhaps the most striking characteristic of [Bem’s] study is that [it meets] the current standards of experimental psychology. The implication is that it is the standards and practices of the field that are not operating properly (p. 155).” Similarly, LeBel & Peters (2011) remark that “...[i]t is precisely because Bem’s report is of objectively high quality that it is diagnostic of potential problems with MRP [Modal Research Practice].... Bem has put empirical psychologists in a difficult position: forced to consider either revising beliefs about the fundamental nature of time and causality or revising beliefs about the soundness of MRP (p. 371).”

LeBel and Peters conclude by recommending that we should put a stronger emphasis on replication. We agree. Rather than continuing to debate Bem’s original experiments, we seek in our meta-analysis to answer the one question that most decisively trumps such disputes: Can independent investigators replicate the original experiments?

## Method

The methodology and reporting of results comply with the Meta-Analysis Reporting Standards ( APA, 2008). Additional materials needed to replicate our results independently can be found at http://figshare.com/articles/Meta-analysis_Implicit_Behavioral_Anticipation/903716.

### Retrieval and coding of experiments

As noted above, the archival summary publication of Bem’s experiments appeared in 2011, but he had begun his first experiments as early as 2000, and began reporting results soon thereafter at departmental colloquia and annual meetings of the Parapsychological Association ( Bem, 2003; Bem, 2005; Bem, 2008). Simultaneously he made materials available to anyone expressing an interest in trying to replicate the experiments. As a result, attempted replications of the experiments began to appear as early as 2001 (as reported in Moulton & Kosslyn, 2011).

No presentiment experiments are included in our database because, as noted above, a meta-analysis of those has already been published ( Mossbridge *et al.*, 2012). We have, however, included 19 attempted replications of Bem’s Retroactive-Facilitation-of Recall experiment that had been previously meta-analyzed by Galak *et al.* (2012) because 8 additional replication studies of that protocol have been reported since then. (This was the only protocol included in Galak *et al.*’s. meta-analysis.)

Although the individual-difference variable of “stimulus seeking” emerged as a significant correlate of psi performance in several of Bem’s original experiments, we have not analyzed that variable in the present meta-analysis because too few of the replications reported on it—especially those that modified Bem’s original protocol.

Co-authors PT, TR, and MD conducted a search for all potentially relevant replications that became available between the year 2000 and September of 2013. These included unpublished reports as well as peer-reviewed, published articles in mainstream psychological journals; specialized journals; proceedings from conferences; and relevant studies found in Google Scholar, PubMed and PsycInfo. The same set of keywords— *Bem, feeling the future, precognition*— was used for all searches, and no MESH terms or Boolean operators were used. Using email and academia.edu, they also contacted known psi researchers and mainstream researchers who had expressed an interest in replicating Bem’s experiments. Of the ninety-three experiments retrieved, two were eliminated because they were severely underpowered: the first had only one participant; the second had nine ( Snodgrass, 2011). A third experiment, reporting positive results, rested on several post-hoc analyses, and so we deemed it too exploratory to include in the meta-analysis ( Garton, 2010). The final database thus comprises 90 experiments.

Co-authors PT and TR independently coded and categorized each study with respect to the following variables: a) type of effect(s) tested; b) number of participants enrolled in the study; c) descriptive or inferential statistics used to calculate measures of effect size; d) whether the replication had been conducted before or after the January, 2011 ( *Online First*) publication of Bem’s original experiments; e) whether or not the experiment had been peer-reviewed; and f) type of replication.

For this last variable, each experiment was categorized into one of three categories: an exact replication of one of Bem’s experiments (31 experiments), a modified replication (38 experiments), or an independently designed experiment that assessed the ability to anticipate randomly-selected future events in some alternative way (11 experiments). To qualify as an exact replication, the experiment had to use Bem’s software without any procedural modifications other than translating on-screen instructions and stimulus words into a language other than English if needed. The eleven experiments that had not been designed to replicate any of Bem’s experiments included five retroactive-priming experiments and six retroactive-practice experiments.

Percentages of agreement for each of the coding variables ranged from a minimum of 90% for the statistical data to 100% for the classification into one of the three categories of experiments. Discrepancies in coding were resolved by discussion between PT and TR.

### Frequentist analysis

All the main inferential statistics, weighted effect-size point estimations with corresponding 95% Confidence Intervals, and combined *z* values were calculated using the Comprehensive Meta-Analysis software v.2 by Borenstein *et al.* (2005). Effect sizes (Hedges’ *g*) and their standard errors were computed from *t* test values and sample sizes. (Hedges’ *g*, is similar to the more familiar *d* [ Cohen, 1988], but pools studies using *n* - 1 for each sample instead of *n*. This provides a better estimate for smaller sample sizes.) When *t* test values were not available, we used the effect sizes reported by the authors or estimated them from the descriptive statistics. When more than one dependent variable was measured, a single effect size was calculated by averaging the effect sizes obtained by the different *t* values.

Heterogeneity within each set of experiments using a particular protocol (e.g., the set of retroactive priming experiments) was assessed using I ^2^ ( Huedo-Medina *et al.*, 2006). It estimates the percent of variance across studies due to differences among the true effect sizes. If all the studies are methodologically identical and the subject samples are very similar, then I ^2^ will be small (< 25%) and a fixed-effect model analysis is justified; otherwise a random-effects model is used ( Borenstein *et al.*, 2009).

A fixed-effect model assumes that all the studies using a particular protocol have the same true effect size and that the observed variance of effect sizes across the studies is due entirely to random error within the studies. The random-effects model allows for the possibility that different studies included in the analysis may have different true effect sizes and that the observed variation reflects both within-study and between-study sampling error.

### Bayesian analysis

A model comparison Bayesian analysis of an experiment pits a specified experimental hypothesis (H _1_) against the null hypothesis (H _0_) by calculating the odds that H _1_ rather than H _0_ is true— *p*(H _1_)/ *p*(H _0_)—or the reverse. The analysis assumes that each person comes to the data with a subjective prior value for these odds and then adjusts them on the basis of the data to arrive at his or her posterior odds. A Bayesian analysis can be summarized by a number called the Bayes Factor (BF), which expresses the posterior odds independent of any particular individual’s prior odds. For example, a BF of 3 indicates that the observed data favor the experimental hypothesis over the null hypothesis by a ratio of 3:1. The posterior odds for a particular individual can then be calculated by multiplying his or her prior odds by BF. For example, a mildly psi-skeptical individual might initially assign complementary probabilities of .2 and .8 to H _1_ and H _0_, respectively, yielding prior odds of .25. If BF = 3 then the Bayesian formula indicates that this individual’s posterior odds should be .75. If BF were to exceed 4, then the posterior odds *p*(H _1_)/ *p*(H _0_) would exceed 1, implying that this individual now favors the experimental hypothesis over the null.

Jeffreys (1998) has suggested the following verbal labels for interpreting BF levels of *p*(H _1_)/ *p*(H _0_):

BF = 1 – 3:        Worth no more than a bare mention
BF = 3 – 10:      Substantial evidence for H _1_
BF = 10 – 30:    Strong evidence for H _1_
BF = 30 – 100:  Very Strong evidence for H _1_
BF > 100:          Decisive evidence for H _1_

To perform a Bayesian analysis, one must also specify a prior probability distribution of effect sizes across a range for both H _0_ and H _1_. Specifying the effect size for H _0_ is simple because it is a single value of 0, but specifying H _1_ requires specifying a probability distribution across a range of what the effect size might be if H _1_ were in fact true. This specification can strongly impact the subsequent estimates of BF and, in fact, was the major disputed issue in the debate over Bem’s original experiments ( Bem *et al.*, 2011; Rouder & Morey, 2011; Wagenmakers *et al.*, 2011).

For purposes of meta-analysis, Rouder & Morey (2011) argue that one should use the Jeffrey, Zellner and Siow (JZS) prior probability distribution (see, also, Bayarri & Garcia-Donato, 2007). That distribution is designed to minimize assumptions about the range of effect sizes and, in this sense, constitutes what is known as an “objective” prior ( Rouder *et al.*, 2009). Moreover, the resulting BF is independent of the measurement scale of the dependent variable, is always finite for finite data, and is consistent in the sense that as sample size increases, BF grows to infinity if the null is false and shrinks to zero if it is true—a consistency that does not obtain for *p* values. Researchers can also incorporate their expectations for different experimental contexts by tuning the scale of the prior on effect size (designated as *r*). Smaller values of *r* (e.g., 0.1) are appropriate when small effects sizes are expected; larger values of *r* (e.g., 1.0) are appropriate when large effect sizes are expected. As *r* increases, BF provides increasing support for the null.

For these several reasons, we have adopted the JZS prior probability distribution for our Bayesian analysis. For the estimation of Bayes Factors, we used the meta.ttest function of the BayesFactor package ( Morey & Rouder, 2014). In the expectation that the effect size will be small, we set *r* = 0.1. To estimate the overall effect size and τ ^2^, a measure of between-studies variance, we employed the DiMaggio (2013) script, which uses the R2jags package to run the “BUGS” program (Bayesian Analysis Using Gibb’s Sampling). This provides a Monte Carlo Markov Chain simulation approach to parameter estimation using a normally distributed prior with a mean of 0.1 and a wide variance of 10 ^5^. The program chooses samples using either Gibbs or Metropolis Hasting algorithms. Because this is a simulation-based approach, we repeated many draws or iterations and evaluated whether the chain of sample values converged to a stable distribution, which was assumed to be the posterior distribution in which we are interested.

We ran two 20,000 Markov Chain Monte Carlo iterations, each starting with different and dispersed initial values for the model. We based our results on the final 20,000 iterations and assessed whether the chain of values had converged to a stable posterior distribution by monitoring and assessing a graph of the chain and by calculating the Brooks Gelman and Rubin statistic, a tool within the CODA package of R programs for this purpose. The results are presented as mean values of the posterior distributions and their 95% credible intervals (CrI).

## Results and discussion

The complete database comprises 90 experiments conducted between 2001 and 2013. These originated in 33 different laboratories located in 14 countries and involved 12,406 participants. The full database with corresponding effect sizes, standard errors, and category assignments is presented in Table S1 along with a forest plot of the individual effect sizes and their 95% confidence intervals.

Table S1 Experiments in the meta-analysis, N, task type, effect size, standard error, peer-review and replication classifications ( Tressoldi *et al.*, 2015). Click here for additional data file. Copyright: © 2016 Bem D et al. 2016 Data associated with the article are available under the terms of the Creative Commons Zero "No rights reserved" data waiver (CC0 1.0 Public domain dedication).

The first question addressed by the meta-analysis is whether the database provides overall evidence for the anomalous anticipation of random future events. As shown in the first and second rows of Table 1, the answer is yes: The overall effect size (Hedges’ *g*) is 0.09, combined *z* = 6.33, *p* = 1.2 × 10 ^-10^. The Bayesian BF value is 5.1 × 10 ^9^, greatly exceeding the criterion value of 100 that is considered to constitute “decisive evidence” for the experimental hypothesis ( Jeffreys, 1998). Moreover, the BF value is robust across a wide range of the scaling factor *r*, ranging from a high value of 5.1 × 10 ^9^ when we set *r* = 0.1 to a low value of 2.0 × 10 ^9^ when *r* = 1.0.

**Table 1.** Meta-analytic results for all experiments and for independent replications of Bem’s experiments.

	Number of experiments	Number of participants	Effect size (Hedges’ *g*)	95%CI or Crl	Combined *z* or Bayes factor	*p* (One-tailed)	I ^2^	*τ* ^2^
All experiments ^a^ Bayesian analysis	90	12,406	0.09 0.08	[0.06, 0.11] [0.02, 0.15]	*z* = 6.33 BF = 5.1×10 ^9^	1.2 × 10 ^-10^	41.4	.005 .028
Independent replications ^b^ Bayesian analysis	69	10,082	0.06 0.07	[0.03, 0.09] [0.01, 0.14]	*z* = 4.16 BF = 3,853	1.2 × 10 ^-5^	36.1	.004 .035
Exact replications Modified replications	31 38	2,106 7,976	0.08 0.05	[0.02, 0.13] [0.02, 0.09]	*z* = 2.90 *z* = 3.00	.0018 .0013	31.7 38.9	.007 .004
Pre-2011 replications Post-2011 replications	30 39	2,193 7,889	0.09 0.05	[0.04, 0.15] [0.02, 0.08]	*z* = 3.20 *z* = 2.88	.0007 .004	39.5 32.3	.009 .003
Peer reviewed Not peer reviewed	35 34	7,477 2,605	0.06 0.06	[0.02, 0.10] [0.02, 0.10]	*z* = 2.93 *z* = 3.21	.0017 .0007	51.4 8.7	.001 .006

*Note*. In a Bayesian analysis, the analogue to the 95%CI is Crl, “credible intervals of the posterior distributions.” I ^2^ is an estimate of the percent of variance across studies due to differences among the true effect sizes. τ ^2^ is the between-studies variance.

^a^ Assuming a null ES of .01 and a variance of .005 (the observed variance, τ ^2^, in the random-effects model), the statistical power of this meta-analysis is 0.95 ( Hedges & Pigott, 2001).

^b^ These analyses exclude Bem’s own experiments and the eleven experiments that had not been designed as replications of those experiments.

The second question is whether independent investigators can successfully replicate Bem’s original experiments. As shown in the third and fourth rows of Table 1, the answer is again yes: When Bem’s experiments are excluded, the combined effect size for attempted replications by other investigators is 0.06, *z* = 4.16, *p* = 1.1 × 10 ^-5^, and the BF value is 3,853, which again greatly exceeds the criterion value of 100 for “decisive evidence.” A robustness analysis shows that the BF value ranges from the 3,853, quoted above, when the *r* parameter is set to .01, to 992 when *r* is set to 1.0.

The fifth and sixth rows of Table 1 show that the mean effect sizes of exact and modified replications are each independently significant and not significantly different from each other (Mean diff = 0.025; 95% CI [-0.04, 0.09]; *z* = 0.87, *ns*).

The seventh and eighth rows show that the mean effect sizes of replications conducted before and after the January, 2011 (online) publication of Bem’s article are each independently significant and not significantly different from each other (Mean diff = 0.042; 95% CI [.02, 0.10]; *z* = 0.37, *ns*).

And finally, the bottom two rows of Table 1 show that the mean effect sizes of peer reviewed and not-peer-reviewed replications are each independently significant and identical to each other.

Table 2 displays the meta-analysis of the complete database as a function of experiment type and divided post-hoc into fast-thinking and slow-thinking protocols.

**Table 2.** Meta-analytic results as a function of protocol and experiment type.

Experiment Type	Number of experiments	Number of participants	Effect size	95%CI	Combined *z*	*p* (One-tailed)	I ^2^
**Fast-thinking protocols**						
Precognitive detection of reinforcement	14	863	0.14 ^a^	[0.08, 0.21]	4.22	1.2 × 10 ^-5^	19.0
Precognitive avoidance of negative stimuli	8	3,120	0.09	[0.03, 0.14]	3.10	.002	50.5
Retroactive priming	15	1,154	0.11	[0.03, 0.21]	2.85	.003	42.0
Retroactive habituation	20	1,780	0.08 ^a^	[0.04, 0.13]	3.50	.0002	24.6
Retroactive practice	4	780	0.11 ^a^	[0.04, 0.18]	3.03	.002	00.0
**All fast-thinking experiments**	**61**	**7,697**	**0.11**	**[0.08, 0.14]**	**7.11**	**5.8 × 10 ^-13^**	31.6
**Slow-thinking protocols**							
Retroactive facilitation of practice on recall	27	4,601	0.04	[-0.01, 0.09]	1.66	.10	38.3
Retroactive facilitation of practice on text reading speed	2	108	-0.10	[-0.40, 0.20]	-0.65	.51	61.0
**All slow-thinking experiments**	**29**	**4,709**	**0.03**	**[-0.01, 0.08]**	**1.38**	**.16**	**39.7**

^a^ Fixed-effect model

As shown in Table 2, fast-thinking protocols fared better than slow-thinking protocols: Every fast-thinking protocol individually achieved a statistically significant effect, with an overall effect size of 0.11 and a combined *z* greater than 7 sigma. In contrast, slow-thinking experiments achieved an overall effect size of only 0.03, failing even to achieve a conventional level of statistical significance ( *p* = .16).

One possible reason for the less successful performance of the slow-thinking experiments is that 12 of the 27 attempted replications of Bem’s retroactive facilitation of recall experiment were modified replications. The 15 exact replications of that protocol yielded an overall effect size of 0.08, but the 12 modified replications yielded a null effect size (-0.00). For example, Galak *et al.* (2012) used their own software to conduct seven of their 11 modified replications in which 87% of the sessions (2,845 of 3,289 sessions) were conducted online, thereby bypassing the controlled conditions of the laboratory. These unsupervised sessions produced an overall effect size of -0.02. Because experiments in a meta-analysis are weighted by sample size, the huge *N* of these online experiments substantially lowers the mean effect size of the replications: When the online experiments are removed, the mean ES for this protocol rises to 0.06 [0.00, 0.12]; *z* = 1.95, *p* = .05.

Nevertheless, we still believe that it is the fast/slow variable itself that is an important determinant of the lower success rate of the slow-thinking experiments. In particular, we suspect that fast-thinking protocols are more likely to produce evidence for psi because they prevent conscious cognitive strategies from interfering with the automatic, unconscious, and implicit nature of psi functioning ( Carpenter, 2012). This parallels the finding in conventional psychology that mere exposure effects are most likely to occur when the exposures are subliminal or incidental because the participant is not aware of them and, hence, is not prompted to counter their attitude-inducing effects ( Bornstein, 1989).

Finally, Table 2 reveals that the clear winner of our meta-analytic sweepstakes is the precognitive detection of erotic stimuli (row 1), the time-reversed version of psychology’s time-honored Law of Effect. The fourteen experiments using that protocol— conducted in laboratories in four different countries—achieve a larger effect size (0.14), a larger combined *z* (4.22), and a more statistically significant result ( *p* = 1.2 × 10 ^-5^) than any other protocol in the Table. This protocol was also the most reliable: If we exclude the three experiments that were not designed to be replications of Bem’s original protocol, 10 of the 11 replication attempts were successful, achieving effect sizes ranging from 0.12 to 0.52. The one exception was a replication failure conducted by Wagenmakers *et al.* (2012), which yielded a non-significant effect in the unpredicted direction, ES = -0.02, *t*(99) = -0.22, *ns*. These investigators wrote their own version of the software and used a set of erotic photographs that were much less sexually explicit than those used in Bem’s experiment and its exact replications.

The results of our meta-analysis do not stand alone. As we noted in the introduction, Bem’s experiments can be viewed as conceptual replications of the presentiment experiments in which participants display physiological arousal to erotic and negative photographs a few seconds before the photographs are selected and displayed ( Mossbridge *et al.*, 2012). The parallel is particularly close for the two protocols testing the precognitive detection of erotic stimuli and the precognitive avoidance of negative stimuli (Protocols 1 and 2 in Table 2). Together those two protocols achieve a combined effect size of 0.11, *z* = 4.74, *p* = 1.07 × 10 ^-6^.

## File-drawer effects: Selection bias

Because successful studies are more likely to be published than unsuccessful studies—the file-drawer effect—conclusions that are drawn from meta-analyses of the known studies can be misleading. To help mitigate this problem, the Parapsychological Association adopted the policy in 1976 of explicitly encouraging the submission and publication of psi experiments regardless of their statistical outcomes. Similarly, we put as much effort as we could in locating unpublished attempts to replicate Bem’s experiments by contacting both psi and mainstream researchers who had requested his replication packages or had otherwise expressed an interest in replicating the experiments. As we saw in Table 1, this all appears to have had the desired effect on the current database: Peer-reviewed experiments yielded the same results as experiments that were not peer-reviewed.

There are also several statistical techniques for assessing the extent to which the absence of unknown studies might be biasing a meta-analysis. We consider nine of them here.

## Fail-safe calculations

One of the earliest of these techniques was the calculation of a “Fail-Safe *N*,” the number of unknown studies averaging null results that would nullify the overall significance level of the database if they were to be included in the meta-analysis ( Rosenthal, 1979). The argument was that if this number were implausibly large, it would give us greater confidence in the conclusions based on the known studies. The Rosenthal Fail-Safe *N*, however, has been criticized as insufficiently conservative because it does not take into account the likely possibility that unpublished or unretrieved studies might well have a mean non-zero effect in the unpredicted direction. Thus the estimate of the Fail-Safe *N* is likely to be too high. (For the record, the Rosenthal Fail-Safe *N* for our database is greater than 1,000.)

An alternative approach for estimating a Fail-Safe *N* focuses on the effect size rather than the *p* value ( Orwin, 1983). The investigator first specifies two numbers: The first is an average effect size for missing studies which, if added to the database, would bring the combined effect size under a specified “trivial” threshold—the second number that must be specified. If we set the mean effect size of missing studies at .001 and define the threshold for a “trivial” effect size to be .01, then the Orwin Fail-Safe *N* for our database is 544 studies. That is, there would have to be 544 studies missing from our database with a mean effect size of .001 to reduce its overall effect size to .01.

## Correlations between study size and effect size

Another set of indices for assessing selection bias are various correlational measures for assessing the relationship between the size of a study and its effect size. The most direct is the Begg and Mazumdar’s rank correlation test, which simply calculates the rank correlation (Kendall’s tau) between the variances or standard errors of the studies and their standardized effect sizes ( Rothstein *et al.*, 2005). If this correlation is significantly negative, if small underpowered studies have larger effect sizes than larger studies, then there is reason to suspect the presence of publication or retrieval bias in the database. For our database, Kendall’s tau is actually slightly positive: τ = **+**0.10; *z* = 1.40, implying that our database is not seriously biased by a selection bias.

More recent publications (e.g., Jin *et al.*, 2015; Rücker *et al.*, 2011; Schwarzer *et al.*, 2010; Stanley & Doucouliagos, 2014; Stanley & Doucouliagos, 2015) have urged the adoption of more complex indices of selection bias:

1. The Copas method ( Copas, 2013; Schwarzer *et al.*, 2010) is based on two models, the standard random effects model and the selection model, which takes study size into account.
2. The Limit meta-analysis ( Schwarzer *et al.*, 2014) is an extended random effects model that takes account of possible small-study effects by allowing the treatment effect to depend on the standard error.
3. The Precision Effect Test (PET, Stanley, 2008; Stanley & Doucouliagos, 2014) is a variant of the classical Egger regression test ( Sterne & Egger, 2005), which tests the relationship between study size and effect size.
4. The Weighted Least Squares analysis ( Stanley & Doucouliagos, 2015) provides estimates that are comparable to random effects analyses when there is no publication bias and are identical to fixed-effect analyses when there is no heterogeneity, providing superior estimates compared with both conventional fixed and random effects analyses.

Table 3 summarizes the results of applying these four additional tests to our database.

**Table 3.** Copas method, Limit meta-analysis, Precision Effect Test and Weighted least squares results for the overall and the “fast-thinking” database.

Test		Effect size estimate	95%CI
Copas method	Overall	0.08	[0.05, 0.10]
	Fast-thinking	0.07	[0.03, 0.10]
Limit meta-analysis	Overall	0.05	[0.02, 0.08]
	Fast-thinking	0.05	[0.01, 0.10]
Precision Effect Test (PET)	Overall	0.01	[-0.04, 0.05]
	Fast-thinking	0.03	[-0.03, 0.08]
Weighted Least Squares	Overall	0.06	[0.04, 0.09]
	Fast-thinking	0.09	[0.06, 0.12]

As Table 3 shows, three of the four tests yield significant effect sizes estimates for our database after being corrected for potential selection bias; the PET analysis is the only test in which the 95% confidence interval includes the zero effect size. As Sterne & Egger (2005) themselves caution, however, this procedure cannot assign a causal mechanism, such as selection bias, to the correlation between study size and effect size, and they urge the use of the more noncommittal term “small-study effect.”

## Trim and fill

**Figure 1.**
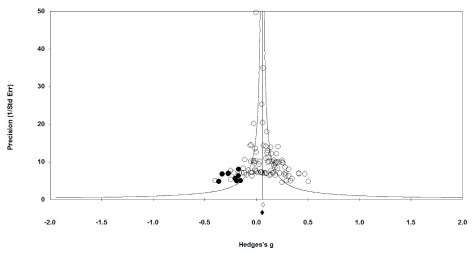
Funnel Plot of the observed studies (white circles) and the imputed missing studies (black circles) under a random-effects model.

Currently the most common method for estimating the number of studies with low effect sizes that might be missing from a database is Duval & Tweedie’s (2000) Trim-and-Fill procedure. It is based on a graphic display of the correlation between sample size and effect size called the “funnel” plot, which plots a measure of sample size on the vertical axis as a function of effect sizes on the horizontal axis. The funnel plot for our database is displayed in Figure 1, which uses the reciprocal of the standard error as the measure of sample size.

If a meta-analysis has captured all the relevant experiments, we would expect the funnel plot to be symmetric: Experiments should be dispersed equally on both sides of the mean effect size. If the funnel plot is asymmetric, with a relatively high number of small experiments falling to the right of the mean effect size and relatively few falling to the left, it signals the possibility that there may be experiments with small or null effects that actually exist but are missing from the database under consideration.

Using an iterative procedure, the trim-and-fill method begins by trimming experiments from the extreme right end of the plot (i.e., the smallest studies with the largest effect sizes) and then calculating a new mean effect size. It then reinserts the trimmed studies on the right and inserts their imputed “missing” counterparts symmetrically to the left of the new mean effect size. This produces a revised, more symmetric funnel plot centered around the newly revised mean effect size. This process continues until the funnel plot becomes symmetric. At that point, the plot is centered around a final corrected estimate of the effect size and displays the number of imputed “missing” experiments to the left of the unbiased mean effect size.

Figure 1 displays the funnel plot for our complete database after it has been modified by the trim-and-fill procedure. The unfilled diamond under the horizontal axis marks the original observed effect size (0.09, see Table 1) and the black diamond marks the corrected estimate of the effect size: 0.07 [0.04, 0.10]. The unfilled circles identify the 90 actual experiments in the meta-analysis; the black circles identify the imputed missing experiments. As Figure 1 shows, there are only eight potentially missing studies. As noted above, the Orwin Fail-Safe estimate of how many missing experiments with low effect sizes would be required to nullify the overall effect size of the database is 544.

## *P*-curve analysis

All the analyses discussed above presume that selection bias is driven by effect-size considerations, but Simonsohn *et al.* (2014a); Simonsohn *et al.* (2014b) have argued that it is actually more likely to be driven by the *p* = .05 significance level. They have also demonstrated empirically that the trim and fill procedure is inadequate for estimating the true effect size present in the database (2014b). In its place, they and other authors ( van Assen *et al.*, 2015) have recently proposed a very different approach called *p-*curve analysis.

*P-*curve is the distribution of significant (p < .05) results among the experiments in a meta-analysis. “It capitalizes on the fact that the distribution of significant *p* values... is a function of the true underlying effect. Researchers armed only with sample sizes and test results of the published findings can correct for publication bias ( Simonsohn *et al.*, 2014b, p. 666).” In addition to assessing selection bias, *p-*curve analysis can also assess the presence of “ *p-*hacking,” questionable practices of selective reporting that illegitimately enable an investigator to claim results that meet the coveted *p* < .05 threshold ( Simonsohn, *et al.*, 2014a; Simonsohn, *et al.*, 2014b).

**Figure 2.**
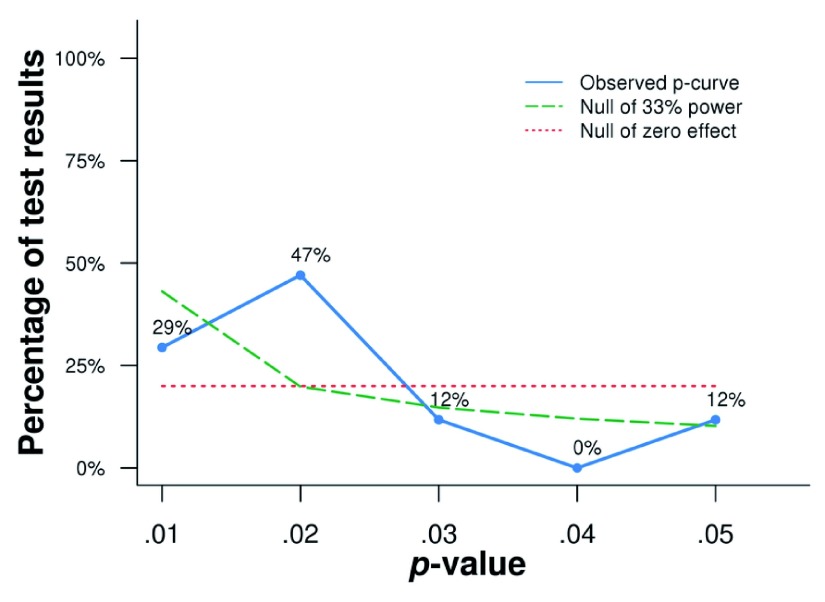
Distribution of the significant *p* values across experiments in the meta-analysis.

In our database, 17 (19%) of the 90 studies reported results that were statistically significant at the .05 level. The solid blue line in Figure 2 displays the *p*-curve distribution of those studies, using Simonsohn *et al’*s (2015) revision of their algorithm.

The dotted horizontal red line (“Null of zero effect”) is the distribution expected if there is no effect in the data. In that case, 5% of the significant *p* values will be below .05, 4% will be below .04, 3% will be below .03, 2% will be below .02, and 1% will be below .01. Thus there will be as many *p* values between .04 and .05 as between .00 and .01, and the shape of the *p*-curve is a uniform, straight horizontal line with 20% of the significant values within each of the 5 intervals on the horizontal-axis. If a genuine non-zero effect exists, however, then *p*-curve’s expected distribution will be right-skewed:

We expect to observe more low significant *p* values ( *p* < .01) than high significant *p* values (.04 < *p* < .05) ( Simonsohn *et al.*, 2014b, pp. 666–667)... A set of significant findings contains evidential value when we can rule out selective reporting as the sole explanation of those findings. Only right-skewed *p*-curves... are diagnostic of evidential value. *P*-curves that are not right-skewed suggest that the set of findings lacks evidential value, and curves that are left-skewed suggest the presence of intense *p*-hacking ( Simonsohn *et al.*, 2014a, p. 535).

The *P*-curve displayed in Figure 2 is significantly right-skewed (Binomial test: *p* = .024; Continuous test: *z* = -1.97, *p* = .024), demonstrating that our database contains evidential value that is not compromised by intense *p*-hacking. A similar conclusion emerges from an analysis using “ *p*-uniform,” the *p*-curve algorithm introduced by Van Assen *et al.* (2015) which directly tests the degree to which the observed curve differs from the “no-effect” uniform distribution. It confirms that there is a significant effect in our database ( *p* = .005) and no evidence for selection bias ( *p* = .86). (If there is a substantial amount of heterogeneity in the meta-analysis, this method should be used as a sensitivity analysis.)

In their updated 2015 discussion of *p*-curve analysis, Simonsohn *et al.* address the special case of “ambitious” *p*-hacking, in which an investigator hacks beyond the .05 level by either dropping higher *p* values (.025 > *p* > .05) or replacing them with lower ones, thereby increasing the odds of a significant right-skew. To test for this, they propose a more stringent test for demonstrating evidential value by requiring that the “half *p*-curve,” the distribution of *p* < .025 results, to be right-skewed as well.

Under this test for “ambitious” *p*-hacking, our database satisfies neither their criterion for evidential value ( *z* = .077, *p* = .22). When that is the case, Simonsohn *et al.* (2014a) propose applying a second test to determine if the observed *p*-curve is significantly flatter than the “Null of 33% power”—depicted by the dashed green line in Figure 2. If it is, then that is affirmative evidence that the *p*-curve actually does lack evidential value. Under that test, our database does not satisfy the criterion for a lack of evidential value either ( *z* = 1.13, *p* = .13). In other words, our database passes the test for not being compromised by “intense” *p*-hacking, but is inconclusive in determining whether “ambitious” *p*-hacking has compromised the half *p*-curve.

In sum, eight of the nine statistical tests we have applied to our database support the conclusion that its overall statistical significance has not been compromised by either selection bias or by *p*-hacking.

## *P*-curve and the true effect size

One of the counterintuitive derivations from *p*-curve analysis—confirmed by extensive simulations—is that when the distribution of significant *p* values is right-skewed, the inclusion of studies with nonsignificant *p* levels ( *p* > .05) in a meta-analysis actually underestimates the true effect size in the database ( Simonsohn *et al.*, 2014b). Based on the Simonsohn *et al. p-*curve analysis, the estimate of the true effect size for our full database is 0.20; for the 69 independent replications, the true effect size is 0.24. These are virtually identical to the mean effect size of Bem’s (2011) original experiments (0.22) and the mean effect size of the presentiment experiments (0.21) ( Mossbridge *et al.*, 2012).

## The complementary merits of exact and modified replications

Our meta-analysis reveals that both exact and modified replications of Bem’s experiments achieve significant and comparable success rates ( Table 1). This is reassuring because the two kinds of replication have different advantages and disadvantages. When a replication succeeds, it logically implies that every step in the replication “worked.” When a replication fails, it logically implies that at least one or more of the steps in the replication failed—including the possibility that the experimental hypothesis is false—but we do not know which step(s) failed. As a consequence, even when exact replications fail, they are still more informative than modified replications because they dramatically limit the number of potential variables that might have caused the failure.

There is, of course, no such thing as a truly exact replication. For example, the experimenter’s attitudes and expectations remain uncontrolled even in a procedurally exact replication, and there are now more than 345 experiments demonstrating that experimenter attitudes and expectations can produce belief-confirming results, even in simple maze experiments with rats as subjects ( Rosenthal & Rubin, 1978).

Exact replications also serve to guard against some of the questionable research practices that can produce false-positive results, such as changing the protocol or experimental parameters as the experiment progresses, selectively reporting comparisons and covariates without correcting for the number examined, and selectively presenting statistical analyses that yielded significant results while omitting other analyses that did not ( Simmons *et al.*, 2011). By defining an exact replication in our meta-analysis as one that used Bem’s experimental instructions, software, and stimuli, we ensure that the experimental parameters and data analyses are all specified ahead of time. In other words, an exact replication is a publicly available, pre-specified protocol that provides many of the same safeguards against false-positive results that are provided by the preregistration of planned experiments.

Despite the merits of exact replications, however, they cannot uncover artifacts in the original protocol that may produce false positive results, whereas suitably modified replications can do exactly that by showing that an experiment fails when a suspected artifact is controlled for. Modified replications can also assess the generality of an experimental effect by changing some of the parameters and observing whether or not the original results are replicated. For example, the one failed replication of the erotic stimulus detection experiment ( Wagenmakers *et al.*, 2012) had substituted mild, non-explicit erotic photographs for the more explicit photographs used in Bem’s original experiment and its exact replications.

As we noted in the introduction, Judd *et al.* (2012) have recently suggested that psychologists should begin to treat stimuli statistically as a random factor the same way we currently treat participants. This would constitute a way of testing the generalizability of results in psychological experiments. This would, however, also represent a major change in current practice in psychology, and none of the experiments in our database treated stimuli as a random factor. Nevertheless, some generality of stimuli used in Bem’s experimental protocols is achieved. In those involving erotic photographs, for example, different stimulus sets are used for men and women and all participants are given the choice of viewing opposite-sex or same-sex erotica. Experiments using words as stimuli (e.g., retroactive priming experiments) were successfully replicated in languages other than English.

The fact that exact and modified replications of Bem’s experiments produced comparable, statistically significant results thus implies generality across stimuli, protocols, subject samples, and national cultures. Moreover, the different protocols can themselves be viewed as conceptual replications of the overarching hypothesis that individuals are capable of anomalously anticipating random future events.

## General discussion

As Bem noted in his original 2011 article, psi is a controversial subject, and most academic psychologists do not believe that psi phenomena are likely to exist. A survey of 1,188 college professors in the United States revealed that psychologists were much more skeptical about psi than respondents in the humanities, the social sciences, or the physical sciences, including physics ( Wagner & Monnet, 1979). Although this survey is now several years old, many psi researchers have observed that psychologists continue to be the most psi-skeptical subgroup of academics.

As Bem further noted, there are, in fact, justifiable reasons for the greater skepticism of psychologists. Although our colleagues in other disciplines would probably agree with the oft-quoted dictum that “extraordinary claims require extraordinary evidence,” we psychologists are more likely to be familiar with the methodological and statistical requirements for sustaining such claims and aware of previous claims that failed either to meet those requirements or to survive the test of successful replication. Even for ordinary claims, our conventional frequentist statistical criteria are conservative: The *p* = .05 threshold is a constant reminder that it is worse to assert that an effect exists when it does not (the Type I error) than to assert that an effect does not exist when it does (the Type II error). (For a refreshing challenge to this view, see Fiedler *et al.*, 2012).

Second, research in cognitive and social psychology over the past 40 years has sensitized us psychologists to the errors and biases that plague intuitive attempts to draw valid inferences from the data of everyday experience (e.g. Gilovich, 1991; Kahneman, 2011). This leads us to give virtually no weight to anecdotal or journalistic reports of psi, the main source cited in the survey by our colleagues in other disciplines as evidence for their more favorable beliefs about psi.

One sobering statistic from the survey was that 34% of psychologists in the sample asserted psi to be impossible, more than twice the percentage of all other respondents (16%). Critics of Bayesian analyses frequently point out the *reductio ad absurdum* case of the extreme skeptic who declares psi or any other testable phenomenon to be impossible. The Bayesian formula implies that for such a person, no finite amount of data can raise the posterior probability in favor of the experimental hypothesis above 0, thereby conferring illusory legitimacy on the most anti-scientific stance. More realistically, all an extreme skeptic needs to do is to set his or her prior odds in favor of the psi alternative sufficiently low so as to rule out the probative force of any data that could reasonably be proffered.

Which raises the following question: On purely statistical grounds, are the results of our meta-analysis strong enough to raise the posterior odds of such a skeptic to the point at which the psi hypothesis is actually favored over the null, however slightly?

An opportunity to calculate an approximate answer to this question emerges from the Bayesian critique of Bem’s original experiments made by Wagenmakers *et al.* (2011). Although they did not explicitly claim psi to be impossible, they came very close by setting their prior odds at 10 ^20^ against the psi hypothesis. As shown in Table 1, the Bayes Factor for our database is approximately 10 ^9^ *in favor* of the psi hypothesis, which implies that our meta-analysis should lower their posterior odds against the psi hypothesis to 10 ^11^. In other words, our “decisive evidence” falls 11 orders of magnitude short of convincing Wagenmakers *et al.* to reject the null. (See a related analysis of their prior odds in Bem *et al.*, 2011.) Clearly psi-proponents have their work cut out for them.

Beyond this Bayesian argument, a more general reason that many psychologists may find a meta-analysis insufficiently persuasive is that the methodology of meta-analysis is itself currently under intense re-examination, with new procedural safeguards (e.g. preregistration of all included studies) and statistical procedures (e.g., treating stimuli as a random factor, *p-*curve analysis) appearing almost monthly in the professional literature. Even though our meta-analysis was conceived and initiated prior to many of these developments, we were able to make use of many of them after the fact, (e.g., *p-*curve analysis) but not others (e.g., preregistration, stimuli treated as a random factor). We thus hope that other researchers will be motivated to follow up with additional experiments and analyses to confirm, disconfirm, or clarify the nature of our findings.

Perhaps the most reasonable and frequently cited argument for being skeptical about psi is that there is no explanatory theory or proposed mechanism for psi phenomena that is compatible with current physical and biological principles. Indeed, this limitation is implied by the very description of psi as “anomalous,” and it provides an arguably legitimate rationale for imposing the requirement that the evidence for psi be “extraordinary.”

We would argue, however, that this is still not a legitimate rationale for rejecting proffered evidence *a priori*. Historically, the discovery and scientific exploration of most phenomena have preceded explanatory theories, often by decades (e.g., the analgesic effect of aspirin; the anti-depressant effect of electroconvulsive therapy) or even centuries (e.g., electricity and magnetism, explored in ancient Greece as early as 600 BC, remained without theoretical explanation until the Nineteenth Century). The incompatibility of psi with our current conceptual model of physical reality may say less about psi than about the conceptual model of physical reality that most non-physicists, including psychologists, still take for granted—but which physicists no longer do.

As is widely known, the conceptual model of physical reality changed dramatically for physicists during the 20th Century, when quantum theory predicted and experiments confirmed the existence of several phenomena that are themselves incompatible with our everyday Newtonian conception of physical reality. Some psi researchers see sufficiently compelling parallels between certain quantum phenomena (e.g., quantum entanglement) and characteristics of psi to warrant considering them as potential mechanisms for psi phenomena (e.g., Broderick, 2007; Radin, 2006). Moreover, specific mechanisms have been proposed that seek to explain psi effects with theories more testable and falsifiable than simple metaphor (e.g., Bierman, 2010; Maier & Buechner, 2015; Walach *et al.*, 2014). A recent collection of these theories is presented in May & Marwaha (2015).

Although very few physicists are likely to be interested in pursuing explanations for psi, the American Association for the Advancement of Science (AAAS) has now sponsored two conferences of physicists and psi researchers specifically organized to discuss the extent to which precognition and retrocausation can be reconciled with current or modified versions of quantum theory. The proceedings have been published by the American Institute of Physics ( Sheehan, 2006; Sheehan, 2011). A central starting point for the discussions has been the consensus that the fundamental laws of both classical and quantum physics are time symmetric:

They formally and equally admit time-forward and time-reversed solutions.... Thus, though we began simply desiring to predict the future from the present, we find that the best models do not require—in fact, do not respect—this asymmetry.... [Accordingly,] it seems untenable to assert that time-reverse causation (retrocausation) cannot occur, even though it temporarily runs counter to the macroscopic arrow of time ( Sheehan, 2006, p. vii).

Ironically, even if quantum-based theories of psi eventually do mature from metaphor to genuinely predictive models, they are still not likely to provide intuitively satisfying descriptive mechanisms for psi because quantum theory itself fails to provide such mechanisms for physical reality. Physicists have learned to live with that conundrum in several ways. Perhaps the most common is simply to ignore it and attend only to the mathematics and empirical findings of the theory—derisively called the “Shut Up and Calculate” school of quantum physics ( Kaiser, 2012).

As physicist and Nobel Laureate Richard Feynman (1994) advised, “Do not keep saying to yourself... ‘but how can it be like that?’ because you will get...into a blind alley from which nobody has yet escaped. Nobody knows how it can be like that (p. 123).”

Meanwhile the data increasingly compel the conclusion that it really *is* like that.

Perhaps in the future, we will be able to make the same statement about psi.

## Data availability

The data referenced by this article are under copyright with the following copyright statement: Copyright: © 2016 Bem D et al.

Data associated with the article are available under the terms of the Creative Commons Zero "No rights reserved" data waiver (CC0 1.0 Public domain dedication). 

*F1000Research*: Dataset 1. Table S1, 10.5256/f1000research.7177.d105136

## Figures and Tables

**Figure S1.** Forest plot of effect sizes. Each blue dot identifies the estimated effect size for that experiment with the corresponding 95% confidence interval. The red vertical line marks the overall effect size based on the random-effects model. Click here for additional data file..
