# Supplementary figures and images for: Feeling the future: A meta-analysis of 90 experiments on the anomalous anticipation of random future events
Source: F1000Res. 2016 Jan 29;4:1188. Originally published 2015 Oct 30. [Version 2] doi: 10.12688/f1000research.7177.2 (PMC4706048; doi:10.12688/f1000research.7177.2)

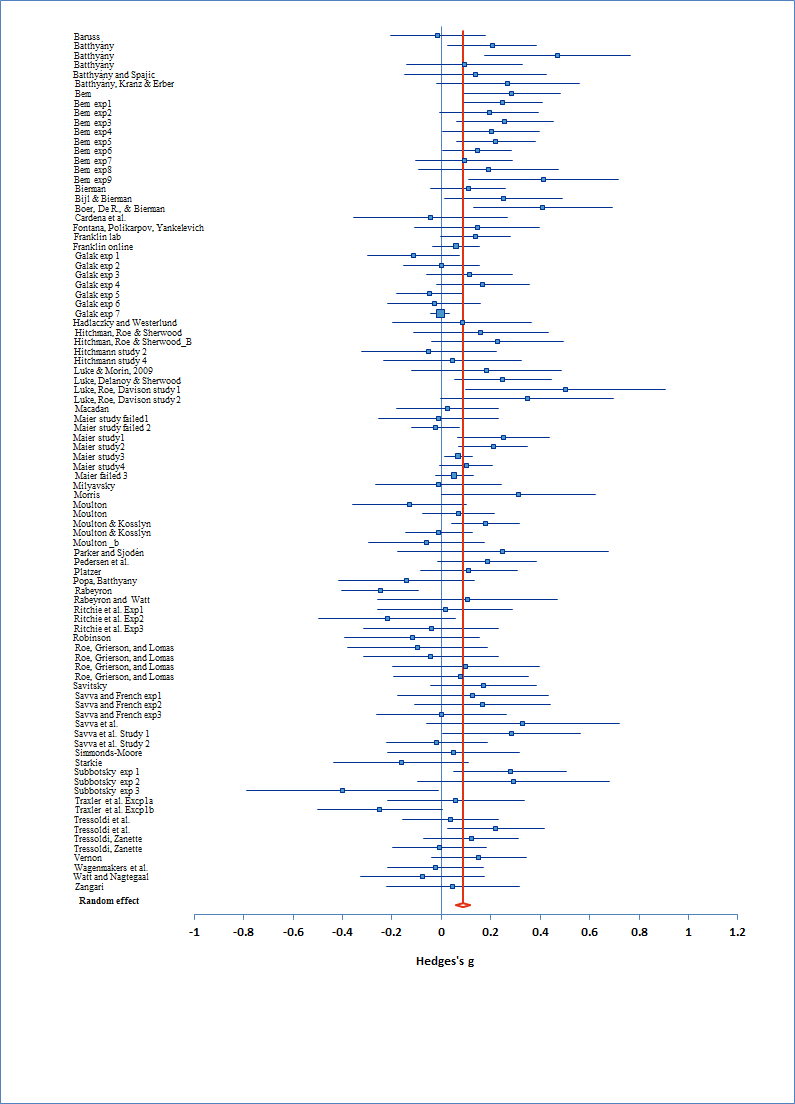

Supplement: Supplementary file 2 [file f1000research-4-8494-s0001.tgz › c1e23b0f-8cfb-44ec-890a-0cf1d1f850cf.tif]
