# Supplementary material for: Feeling the future: A meta-analysis of 90 experiments on the anomalous anticipation of random future events
Source: F1000Res. 2016 Jan 29;4:1188. Originally published 2015 Oct 30. [Version 2] doi: 10.12688/f1000research.7177.2 (PMC4706048; doi:10.12688/f1000research.7177.2)
Supplement: Peer review history

# Peer review: Referee response for version 1

**DOI:** 10.5256/f1000research.7733.r11510

**Published:** 2015-12-21

**Authors:** Paul Grigoriev

**Affiliations:** 1 Department of Medical Physics and Informatics, Crimea State Medical University, Crimea, Ukraine

The research article “Feeling the future: A meta-analysis of 90 experiments on the anomalous anticipation of random future events” by Daryl Bem, Patrizio Tressoldi, Thomas Rabeyron, Michael Duggan raises the fundamental problem: if “nonlocal” anticipation really exists. In his original research Bem (2011) constructed several experimental designs that could indicate time-reversed cognitive and emotional effects similar to well-known and approved in psychology (approach and avoidance, priming, habituation, facilitation of recall).

Bem’s innovation to experimental approach in “psi” research is quite prominent because he started to use several methods based not on so-called force-choice. Thus, implicit drives and motivators started playing a role in results. For example, in experiments on “retroactive approach and avoidance” not the fact of guessing by itself is estimated, but the percentage of pictures with some similar content that may actualize similar emotions and drives in most people.

More generally, it is proved that intuition and anticipation are more effective and frequent in situations with lack of time and/or information for decision-making (Vasilieva, 2006). In special computer games with unpredicted results based on usage of random number generators we can see notable effect in guessing, especially in first 20 attempts (Li, 1992) while interest is high enough.

In present article, when Bem’s results excluded, the effect size became lesser (combined effect size for attempted replications by other investigators is 0.06, z = 4.16, p = 1.1 × 10 ^-5^). I do not think as a majority of sceptics, that Bem could select best results anyhow. For my opinion, it may be because Bem’s participants were the students – mostly young people with higher sex drive according to age and social status. In our recent investigation (Grigoriev, Vasilieva, 2015) we got similar results (to Bem’s) within young men (they guessed erotic photos more frequently), but results were opposite for men of higher age (they “guessed” erotic pictures significantly rarer than expected).

Really, Bem based his research, in particular, on thesis that participants should be attracted by erotic pictures and move away from unpleasant pictures. Thus, generally we follow our drives or motivations when select photos like we could see the “images of the future” or some contented hints from the future. If we even accept this fantastic assumption, we should remember than each individual is characterized by own structure of needs and psychological defenses, more over they depend from actual psychological and physiological state. So, relatively small effect size may be caused also by total averaging of participants. If we select participants to be similar in some respect, for example, men in prison or students under severe risk of expelling from university, we get more effect size (Grigoriev, Vasilieva, 2015), because real majority of participants were in similar and specific stress situations.

Underlying hypothesis about retroactive influence of future events on psyche and physiological state of individual seems to be reasonable in general, although we know nothing about physical mechanisms of such influence except of some metaphors from quantum physics like “nonlocality” on “entanglement”… Still entangled particles are interconnected not only in space, but also in time… Again, there is a place for strong discussion between different physicists. We do not know actually, if entanglement could be enough lasting and existing at high temperatures, and consist enough amount of information within particles like photons, for example.

Nevertheless, an argument that it is not worth to study something, only because nobody knows the mechanism of phenomenon, seems to be improper. At least, on the stage of exploratory researches. Moreover, skeptics’ arguments that anticipation of unpredictable events has no any sense for an organism is unqualified. Theory of functional systems of Pyotr Anokhin (Anokhin, 1973) is one of keystones of biology, like the meaning of anticipation (Lomov and Surkov, 1980; Lomov, 1984) for psychological adaptation.

We should care only about reproducibility. Yes, there are some difficult problems with reproducibility in investigations of psi in general. Although I do not think that retroactive influence of future events is “psi” at all. We do not know delicate physical and psychological mechanism that may work and provide quite small, but significant effect-size. One could offer at least several ways to overcome such weak reproducibility: 1. We should use longitude (during many days and repeats) for every participant and take into account different variables like physiological state and outer influences like weather, social events etc. 2. Parallel registration of processes in nervous system (McCraty, 2004; Mossbridge et al, 2012; Bierman & Scholte, 2002) to distinguish specific patterns in physiological processes when anomalous perception of unpredictable future events is successful. 3. Higher motivation on success or failure avoidance in participants. 4. Taking into account specific needs and state of every participant that may cause differences of unconscious setting to get the specific results.

Of course, this research is still quite odd for traditional psychology and may need some extraordinary evidences. At least authors provide us by enough statistical evidences of effects. The sources of possible artifacts were reviewed on the stages of data collecting, experimental methods and statistical analysis. It is worth to mention, that both Bayesian analysis and effect-size with statistical significance indices are enough high even after elimination of all “suspicious” data.

For my opinion, article may be published, because obtained positive results about possibility of anomalous retroactive influence reflect some unknown nonlocal mechanism that acts through time and can be felt by psyche and/or organism. It sounds weird; but we should remember every revolution in science had started from such odd facts, that incorporated then into the new theory…

And, as for the problem of weak reproducibility of results, the main strategy here should be the searching for the highly reproducible factors that cause variability of phenomenon. When we discover the reasons of nonreproducibility – thus we could understand a structure of phenomenon better.

I have read this submission. I believe that I have an appropriate level of expertise to confirm that it is of an acceptable scientific standard.

# Peer review: Referee response for version 1

**DOI:** 10.5256/f1000research.7733.r11511

**Published:** 2015-12-17

**Authors:** Ina Vitalevna Vasileva

**Affiliations:** 1 Department of General and Social Psychology, Tyumen State University, Tyumen, Russian Federation

The article describes the results of meta-analysis 90 experiments performed to check is whether the anomalous anticipation of random future events exist. These results were obtained in 33 different laboratories located in 14 countries and involved 12.406 participants. Daryl Bem initially constructed and realized 4 types of experiments: 1. Precognitive Approach and Avoidance; 2. Retroactive Priming; 3. Retroactive Habituation; 4. Retroactive Facilitation of Recall. In each type of experiment, an influence of time-reserved effects was checked. An effect-size in researches included is enough to support the hypothesis about anomalous anticipation of random future events.

Online experiments revealed less successful comparing with traditional experiments with experimenter. Authors suppose that lesser effect-size in online-experiments is caused by inability to control all experimental conditions. I believe that it is an adequate explanation of differences in effect in online and laboratory experiments.

Authors discussed a possible influence of experimenter’s belief in psi. It agrees with some hypothetical ideas of “quantum consciousness”. Unfortunately, for three time-reserved experiments (1. Precognitive Approach and Avoidance; 2. Retroactive Priming; 3. Retroactive Habituation) such characteristics as valence and arousal for erotic and unpleasant photographs (The International Affective Picture System) were not described. We could estimate obtained results differential characteristics of participants (gender, age, social status). Particularly, in our recent research (Grigoriev, Vasilieva, 2015) we have obtained the results concerning relationship between such characteristics as a gender, age, satisfaction of basic needs and specific of affective visual stimuli anticipation (similar to “Precognitive Approach and Avoidance” type of experiment described in article and also using stimuli from IAPS) in cohorts of students (men and women), law-abiding and convicted of violent crimes men.

Authors also discussed the differences in effect-size between «fast-thinking» and «slow-thinking» strategies. They suggest that fast/slow variable effects on result of psi. From one side I agree with this experimental fact and conclusion, because «fast-thinking», as authors say, «prevent conscious cognitive strategies», although still suppose that detailed research is necessary to continue in respect to these variables, because participants’ strategies may be caused not only with the speed of operation with stimuli, but also with content of stimuli.

This article may be accepted for indexing because of its proper methodological and methodical level. Enough variables that may affect psi were taken in consideration.

I have read this submission. I believe that I have an appropriate level of expertise to confirm that it is of an acceptable scientific standard.
